# Crystal structure and Hirshfeld surface analysis of 3-phenyl-1-{3-[(3-phenyl­quinoxalin-2-yl)­oxy]prop­yl}-1,2-di­hydro­quinoxalin-2-one

**DOI:** 10.1107/S2056989024001518

**Published:** 2024-02-20

**Authors:** Nadeem Abad, Joel T. Mague, Abdulsalam Alsubari, El Mokhtar Essassi, Abdullah Yahya Abdullah Alzahrani, Youssef Ramli

**Affiliations:** a Laboratory of Medicinal Chemistry, Drug Sciences Research Center, Faculty of Medicine and Pharmacy Mohammed V University in Rabat, Morocco; b Laboratory of Heterocyclic Organic Chemistry, Faculty of Sciences, Mohammed V University, Rabat, Morocco; c Department of Chemistry, Tulane University, New Orleans, LA 70118, USA; d Laboratory of Medicinal Chemistry, Faculty of Clinical Pharmacy, 21 September University, Yemen; e Laboratory of Heterocyclic Organic Chemistry Faculty of Sciences, Mohammed V University, Rabat, Morocco; f Department of Chemistry, Faculty of Science and Arts, King Khalid University, Mohail, Assir, Saudi Arabia; g Mohammed VI Center for Research and Innovation (CM6), Rabat 10000, Morocco; Katholieke Universiteit Leuven, Belgium

**Keywords:** crystal structure, quinoxaline, alkyl­ation, hydrogen bond, π-stacking, Hirshfeld surface analysis

## Abstract

In the title compound, C_31_H_24_N_4_O_2_, the quinoxaline units are distinctly non-planar and twisted end-to-end. In the crystal, C—H⋯O and C—H⋯N hydrogen bonds link the mol­ecules into chains extending along the *a*-axis direction. The chains are linked through π-stacking inter­actions between inversion-related quinoxaline moieties.

## Abstract 2

In the title compound, the quinoxaline units are distinctly non-planar and twisted end-to-end. In the crystal, C—H⋯O and C—H⋯N hydrogen bonds link the mol­ecules into chains extending along the *a*-axis direction. The chains are linked through π-stacking inter­actions between inversion-related quinoxaline moieties.

## 1. Chemical context

The therapeutic and industrial importance of nitro­gen-containing heterocyclic rings has attracted much attention. Among the various classes of nitro­gen-containing heterocyclic compounds, quinoxaline derivatives have an important role in medicinal chemistry and display a broad spectrum of biological and pharmacological activities such as anti­microbial, anti­viral, anti­cancer, anti-inflammatory, anti-diabetic, anti-HIV, anti-tubercular and analgesic activities (Ramli & Essassi, 2015 ▸). Some analogs have been synthesized and evaluated for their industrial properties (*e.g.* Lgaz *et al.*, 2015 ▸). 
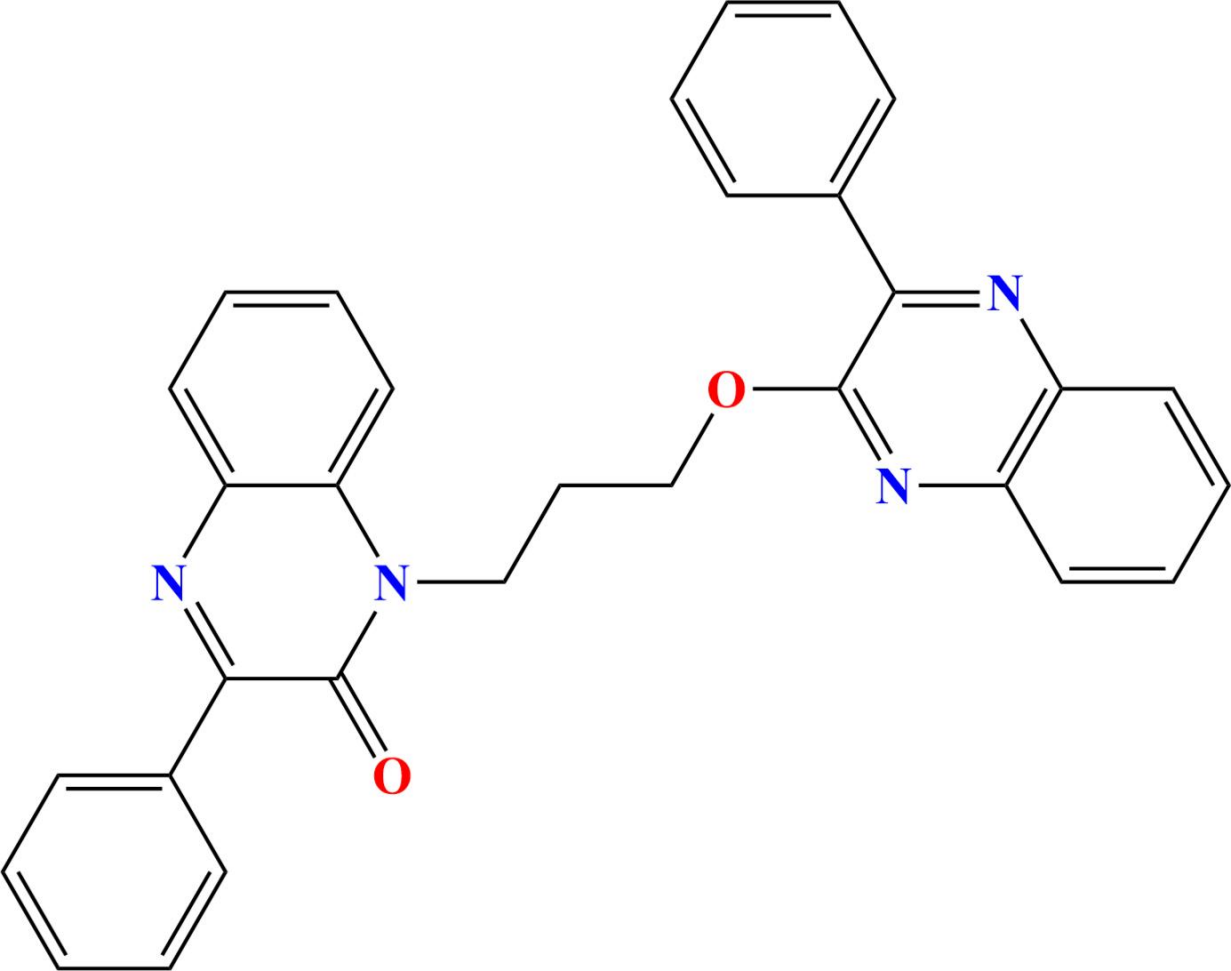


**Figure 1:**
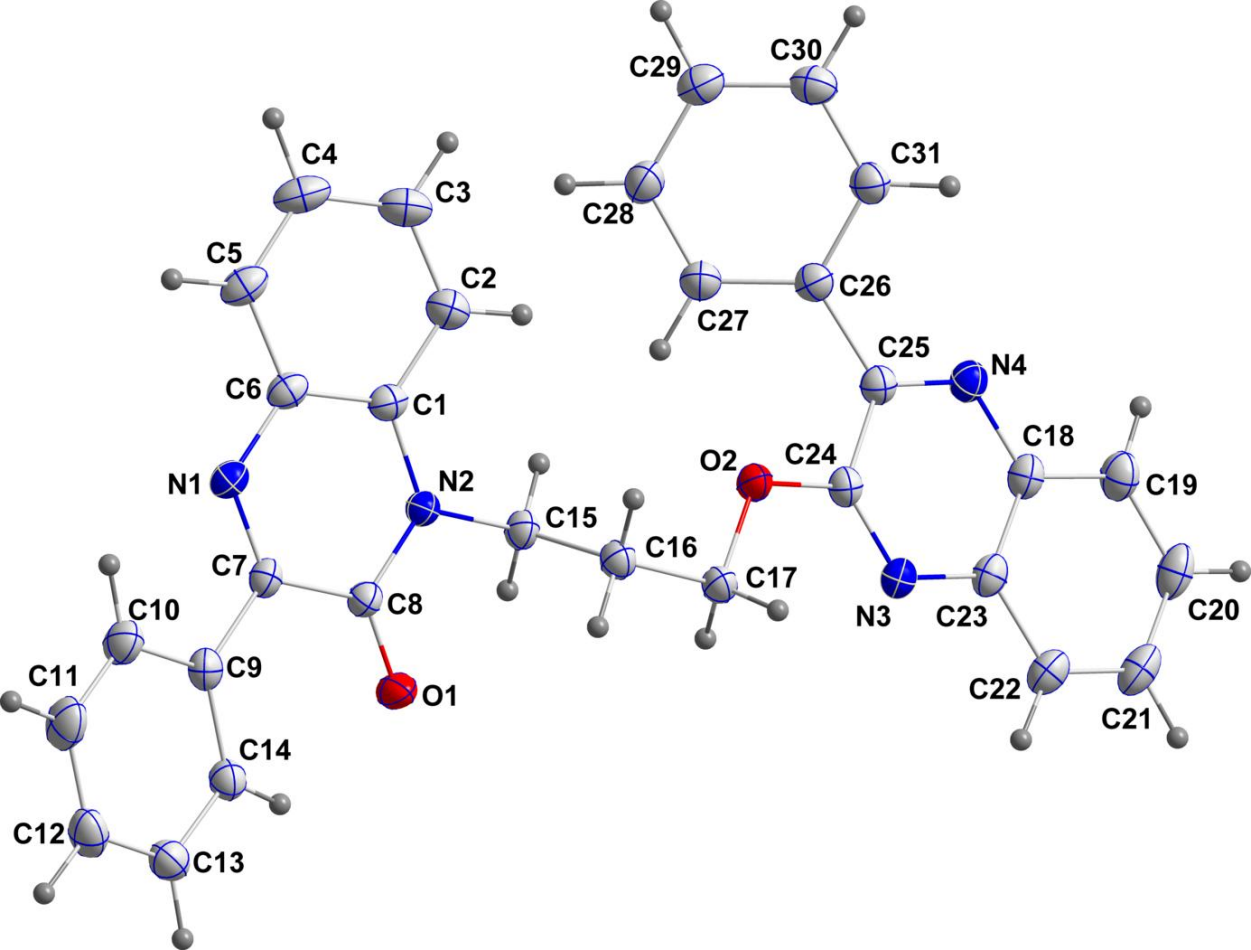
The title mol­ecule with the labeling scheme and 50% probability ellipsoids.

Our inter­est in quinoxalines results from their simple synthesis, and the ease with which X-ray quality crystals can be grown. Following this line of research, and as a continuation of our work in this area (*e.g.* Missioui *et al.*, 2022 ▸), we report herein the synthesis of 3-phenyl-1-{3-[(3-phenyl­quinoxalin-2-yl)­oxy]prop­yl}-1,2-di­hydro­quinoxalin-2-one obtained by an alkyl­ation reaction of 3-phenyl­quinoxalin-2(1*H*)-one using 1,3-di­bromo­propane as an alkyl­ating reagent and sodium hydroxide in the presence of tetra-*n*-butyl­ammonium bromide as catalyst in phase-transfer catalysis. A colorless plate-like specimen of the title compound was used for the X-ray crystallographic analysis (Fig. 1 ▸). A Hirshfeld surface analysis was performed to analyze the inter­molecular hydrogen bonds.

## 2. Structural commentary

Neither quinoxaline unit is planar and in both instances, the heterocyclic ring has atoms deviating by 0.02–0.04 Å from the mean plane. Thus in the pyrazine ring containing N1, atom C7 is 0.0411 (7) Å from the mean plane and C8 is −0.0356 (7) Å from it (r.m.s. deviation of the fitted atoms = 0.0299 Å). The C1–C6 ring is inclined to the above plane by 4.99 (8)° while the dihedral angle subtended with the C9–C14 ring is 11.51 (7)°. The rotational orientation of the former ring is partially determined by the intra­molecular C14—H14⋯O1 hydrogen bond (Table 1 ▸ and Fig. 1 ▸), while that of the latter ring may be influenced by a C27—H27⋯O2 hydrogen bond [H27⋯O2 = 2.448 (13) Å, C27⋯O2 = 2.8791 (14) Å], but with the C27—H27⋯O2 angle being only 106.1 (9)°, this is weak at best. At the other end, the pyrazine ring containing N3 is closer to planarity with displacements from the mean plane being 0.0174 (7) Å (N4) and −0.0195 (7) Å (C25) (r.m.s. deviation of the fitted atoms = 0.0150 Å). Here the dihedral angle to the C18–C23 plane is 2.85 (7)° and that to the C26–C31 ring is 38.75 (4)°. The linker between the quinoxaline units is rather kinked, as seen from the torsion angles in Table 2 ▸.

## 3. Supra­molecular features

**Figure 2:**
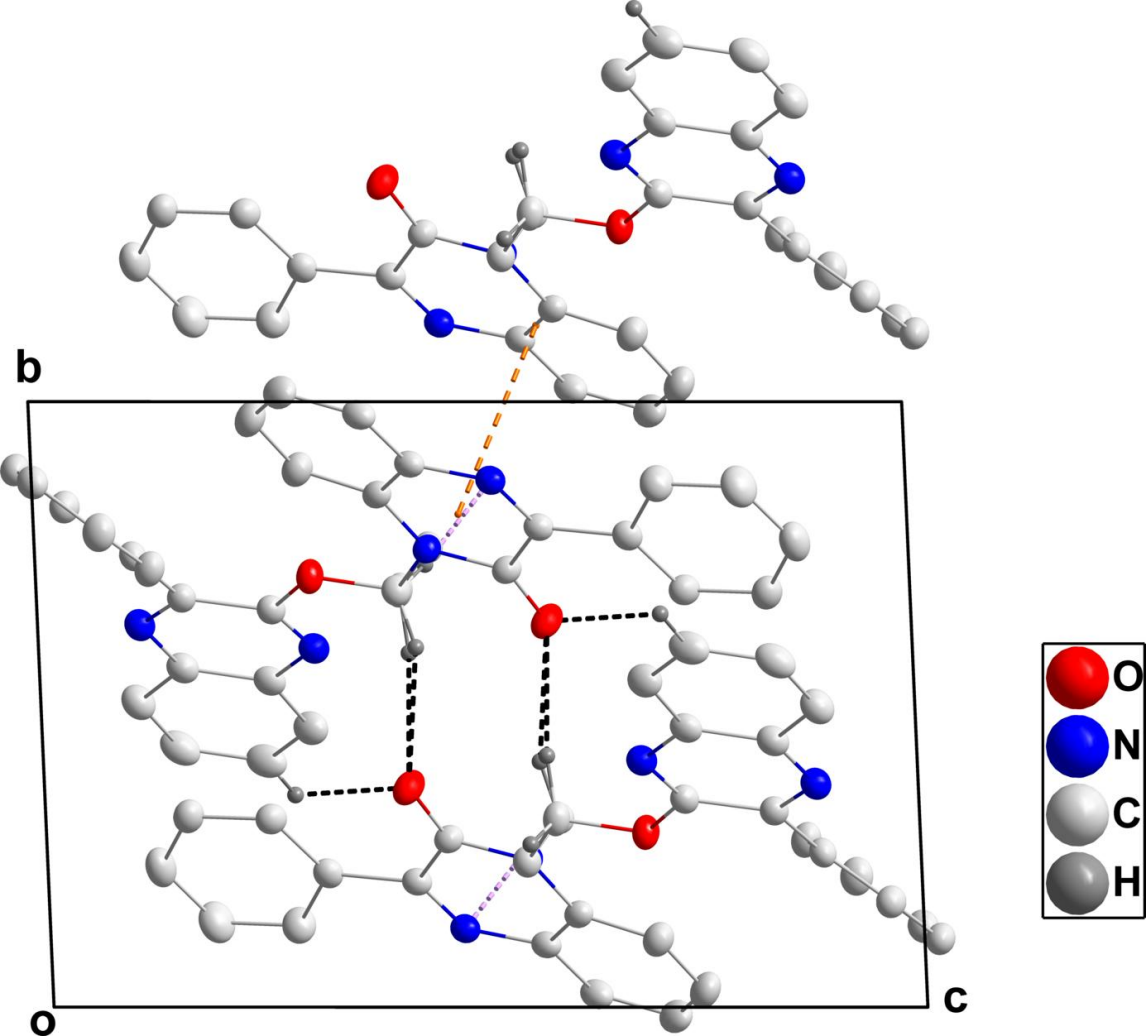
Packing viewed along the *a*-axis direction. C—H⋯O and C—H⋯N hydrogen bonds are indicated by black and light-purple dashed lines, respectively. The π-stacking inter­action is indicated by an orange dashed line.

In the crystal, C15—H15*B*⋯O1, C17—H17*B*⋯O1, C21—H21⋯O1 and C17—H17*A*⋯N1 hydrogen bonds (Table 1 ▸) link the mol­ecules into chains extending along the *a*-axis direction (Figs. 2 ▸ and 3 ▸). The chains are linked through π-stacking inter­actions between inversion-related quinoxaline moieties with centroid–centroid distances of 3.7756 (6) and 3.6440 (7) Å (Figs. 2 ▸ and 3 ▸).

## 4. Hirshfeld surface analysis

**Figure 4:**
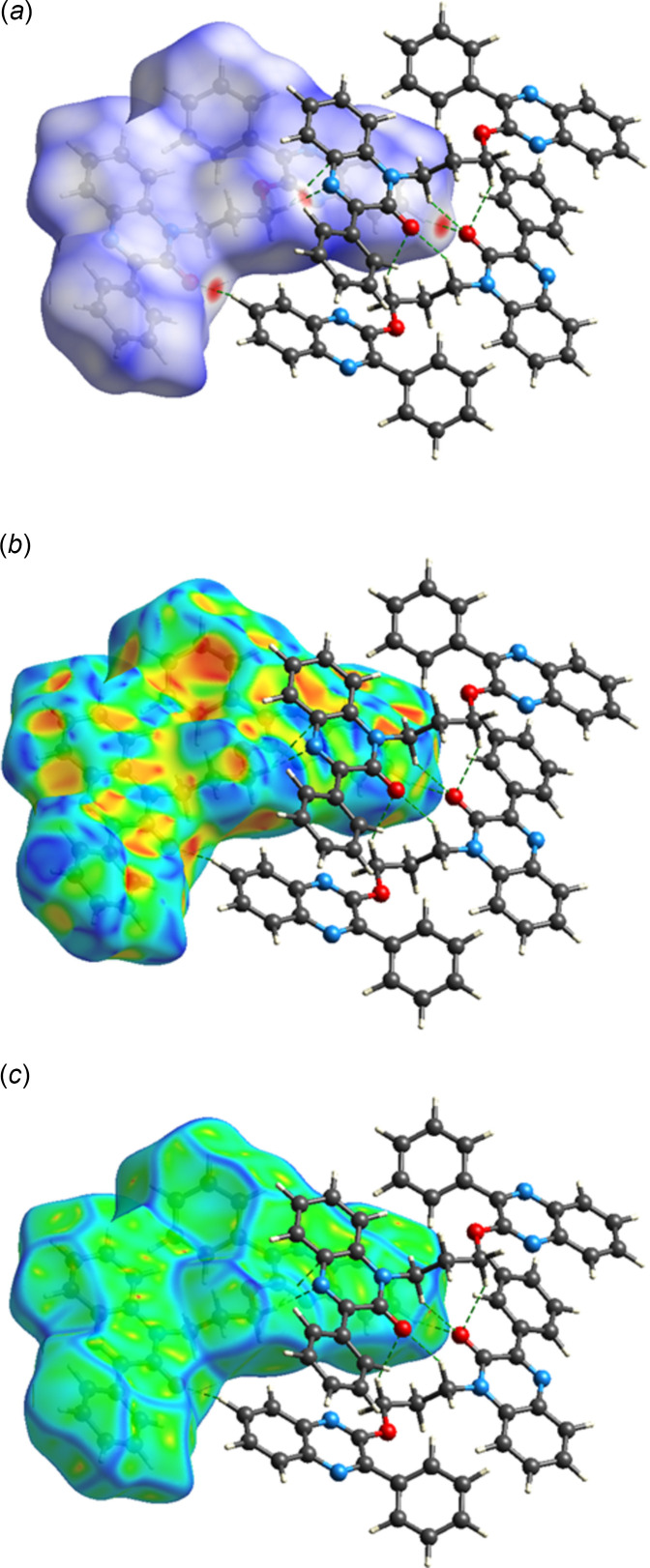
The Hirshfeld surfaces (*a*) *d*_norm_, (*b*) shape index and (*c*) curvedness with three neighboring mol­ecules showing the C—H⋯O and C—H⋯N hydrogen bonds (dashed lines).

**Figure 5:**
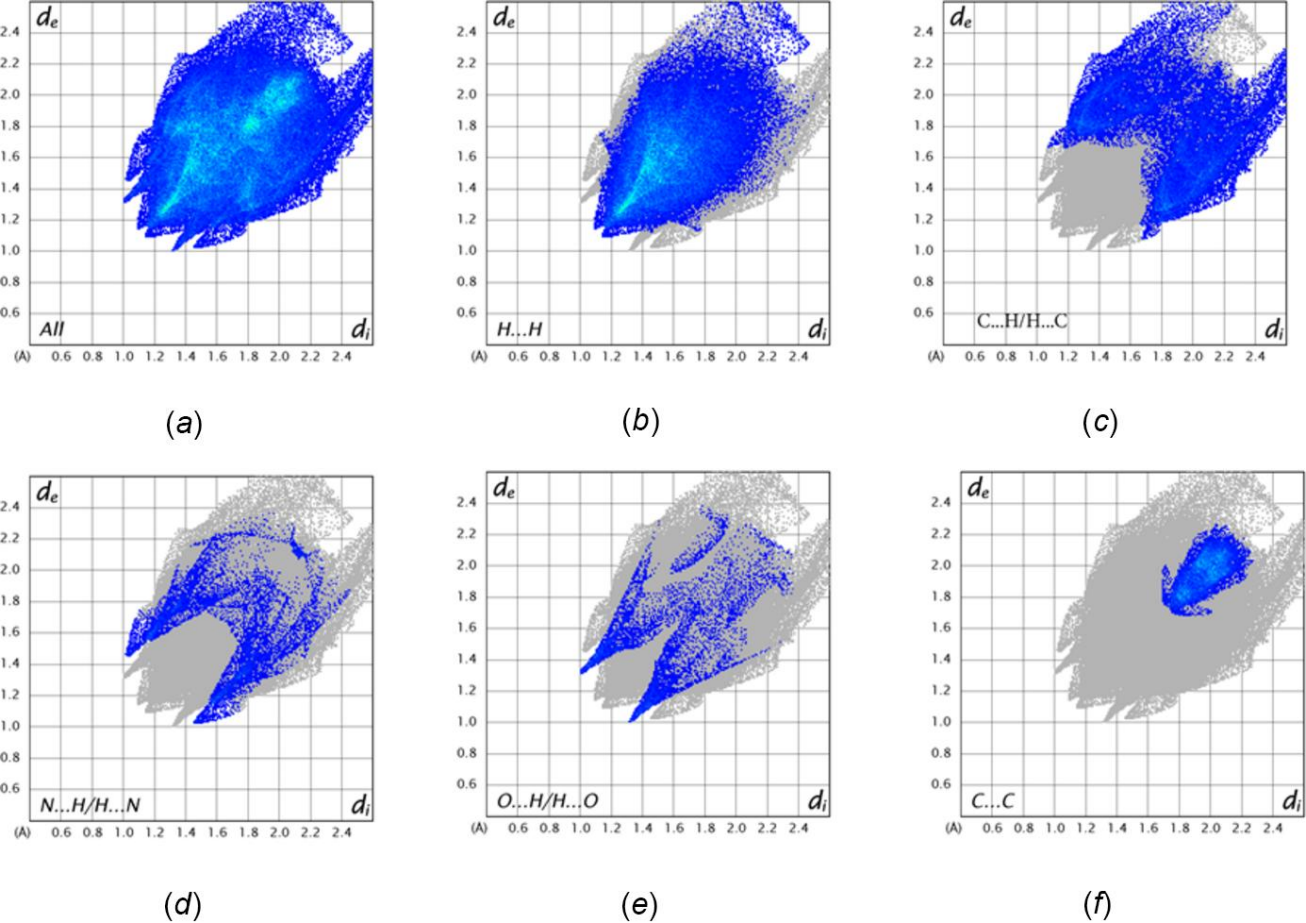
Two-dimensional fingerprint plots showing (*a*) all inter­molecular inter­actions, and delineated into (*b*) H⋯H, (*c*) C⋯H/H⋯C, (*d*) C⋯C, (*e*) N⋯H/H⋯N and (*f*) O⋯H/H⋯O contacts.

The inter­molecular inter­actions in the crystal were qu­anti­fied through a Hirshfeld Surface (HS) analysis using *CrystalExplorer 21.5* (Spackman *et al.*, (2021 ▸). Additional details of the inter­pretation of the results have been published (Tan *et al.*, 2019 ▸). In the standard *d*_norm_ surface (Fig. 4 ▸ *a*) the C—H⋯O and C—H⋯N hydrogen bonds to the closest neighboring mol­ecules are depicted by green dashed lines. In Fig. 4 ▸ *b* (shape-index) and 4*c* (curvedness), the π-stacking inter­actions involving the neighboring mol­ecule that has the most overlap with the surface can be seen. This is particularly evident in Fig. 4 ▸ *c* where the quinoxaline rings are separated by a significant flat region of the surface. A similar flat region appears on the left side of the surface in Fig. 4 ▸ *c*. The overall two-dimensional fingerprint plot, Fig. 5 ▸ *a*, and those delineated into specific inter­molecular inter­action types are shown in Fig. 5 ▸ *b*–*f*. From these, H⋯H contacts account for 51.3% of the total, while C⋯H/H⋯C contribute another 24.2%. The remaining significant contacts are C⋯C (π-stacking, 9.0%), N⋯H/H⋯N (6.5%), O⋯H/H⋯O (5.0%) and C⋯N (π-stacking, 3.5%).

## 5. Database survey

**Figure 6:**
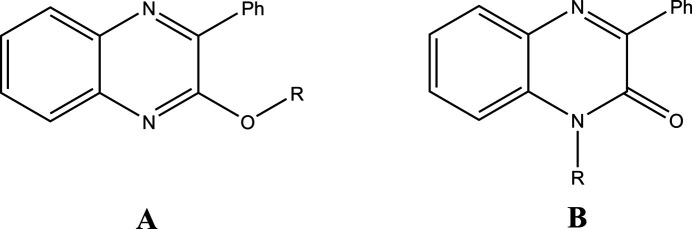
CSD search fragments.

A search of the Cambridge Structural Database (CSD, Version 5.44, updated to November 2023; Groom *et al.*, 2016 ▸) with the search fragment **A** (Fig. 6 ▸, *R* = C) yielded two hits with *R* = benzyl (FACPEI; Abad *et al.*, 2020 ▸) and *R* = (oxazolidin-2-one-3-yl)ethyl (UREREP; Daouda *et al.*, 2011 ▸). In the former, the r.m.s. deviation of the quinoxaline atoms from their mean plane is 0.001 Å, while the phenyl ring is inclined to this plane by 39.32 (5)^o^ and the C—O—C—C torsion angle in the benz­yloxy linker is 97.06 (11)^o^. In the latter, the quinoxaline ring atoms vary from 0.040 (3) to −0.047 (2) Å from the mean plane in one independent mol­ecule and 0.046 (4) to −0.075 (3) Å in the other. The phenyl ring is inclined to the mean quinoxaline plane by 38.44 (14)^o^ in the first and 38.97 (14)^o^ in the second.

Using the fragment **B** (Fig. 6 ▸), fifteen hits were returned with *R* = –(CH_2_)_7_Me (AZAZEC; Abad *et al.*, 2021*d* ▸), Me (BUDMAP; Benzeid *et al.*, 2009*a* ▸), ethyl (1*H*-1,2,3-triazol-1-yl)methyl acetate (ECUCOY; Abad *et al.*, 2022 ▸), –(CH_2_)_2_OC=O)Me (ESUKUB; Abad *et al.*, 2021*a* ▸), (1-hexyl-1*H*-1,2,3-triazol-5-yl)methyl (FOFCIQ; Abad *et al.*, 2023*a* ▸), (oxazolidin-2-one-3-yl)ethyl (IDOSUR; Al Ati *et al.*, 2021 ▸), [3-(4-methyl­phen­yl)-4,5-di­hydro-1,2-oxazol-5-yl]methyl (ILI­RED; Abad *et al.*, 2021*b* ▸), Et (MAGBIJ; Al Ati *et al.*, 2021 ▸), (oxirane-2-yl)methyl (NIBXEE; Abad *et al.*, 2018*a* ▸), benzyl (PUGGII; Benzeid *et al.*, 2009*b* ▸), –(CH_2_)_2_CH_2_OH (RIRBOM; Abad *et al.*, 2018*b* ▸), –(CH_2_)_8_Me (UDAMIZ; Abad *et al.*, 2021*c* ▸), –(CH_2_)_4_Me (UFITEM; Abad *et al.*, 2023*b* ▸), –(CH_2_)_2_CO_2_Et (XEXWIJ; Abad *et al.*, 2018*c* ▸) and allyl (YAJGEX; Benzeid *et al.*, 2011 ▸). Three of these structures feature two independent mol­ecules in the asymmetric unit (see Table 3 ▸). For these last fifteen structures, Table 3 ▸ lists the largest distance of an atom in the quinoxaline moiety from its mean plane (*d*_max_) and the dihedral angle between the mean planes of the quinoxaline moiety and the attached phenyl ring (α). From these, it can be concluded that the deviation from planarity of the quinoxaline rings in the present structure is comparable to that in the related mol­ecules, while the rotation of the phenyl ring out of the plane of the quinoxaline is at the low end of the observed dihedral angles. Also presented in Table 3 ▸ are torsion angles for parts of the related mol­ecules corresponding to N2—C15—C16—C17 in the present structure.

## 6. Synthesis and crystallization

To a solution of 3-phenyl­quinoxalin-2(1*H*)-one (0.5 g, 2.25 mmol) in *N,N*-dimethylformamide (15 ml) were added 1,3-di­bromo­propane (0.12 ml, 1.125 mmol), sodium hydroxide (0.1 g, 2.25 mmol) and a catalytic qu­antity of tetra-*n*-butyl­ammonium bromide. The reaction mixture was stirred at room temperature for 24 h. The solution was filtered and the solvent removed under reduced pressure. The residue obtained was chromatographed on a silica gel column using a hexa­ne/ethyl acetate 9:1 mixture as eluent and the solid obtained upon solvent removal was recrystallized from ethanol to afford colorless plate-like crystals of the title compound. **^1^H NMR** (300 MHz, CDCl_3_) δ ppm: 1.95–2.03 (*quin*, 2H, CH_2_); 3.53 (*t*, 2H, N-CH_2_, *J* = 6Hz); 4.44 (*t*, 2H, O-CH_2_, *J* = 6Hz); 7.30–8.23 (*m*, 18H, CH_arom_). **^13^C NMR** (75 MHz, CDCl_3_) δ ppm: 26.66 (CH_2_); 40.27 (N—CH_2_); 64.28(O—CH_2_); 113.36–130.76 (CH_arom_); 132.47–154.01 (Cq); 154.48 (C—O); 155.22 (C=O); 155.22 (C—O).

## 7. Refinement

Crystal data, data collection and structure refinement details are summarized in Table 4 ▸. Hydrogen atoms were refined isotropically.

## Supplementary Material

Crystal structure: contains datablock(s) global, I. DOI: 10.1107/S2056989024001518/vm2295sup1.cif

Structure factors: contains datablock(s) I. DOI: 10.1107/S2056989024001518/vm2295Isup2.hkl

Supporting information file. DOI: 10.1107/S2056989024001518/vm2295Isup3.cml

CCDC reference: 2332949

Additional supporting information:  crystallographic information; 3D view; checkCIF report

## Figures and Tables

**Figure 3:**
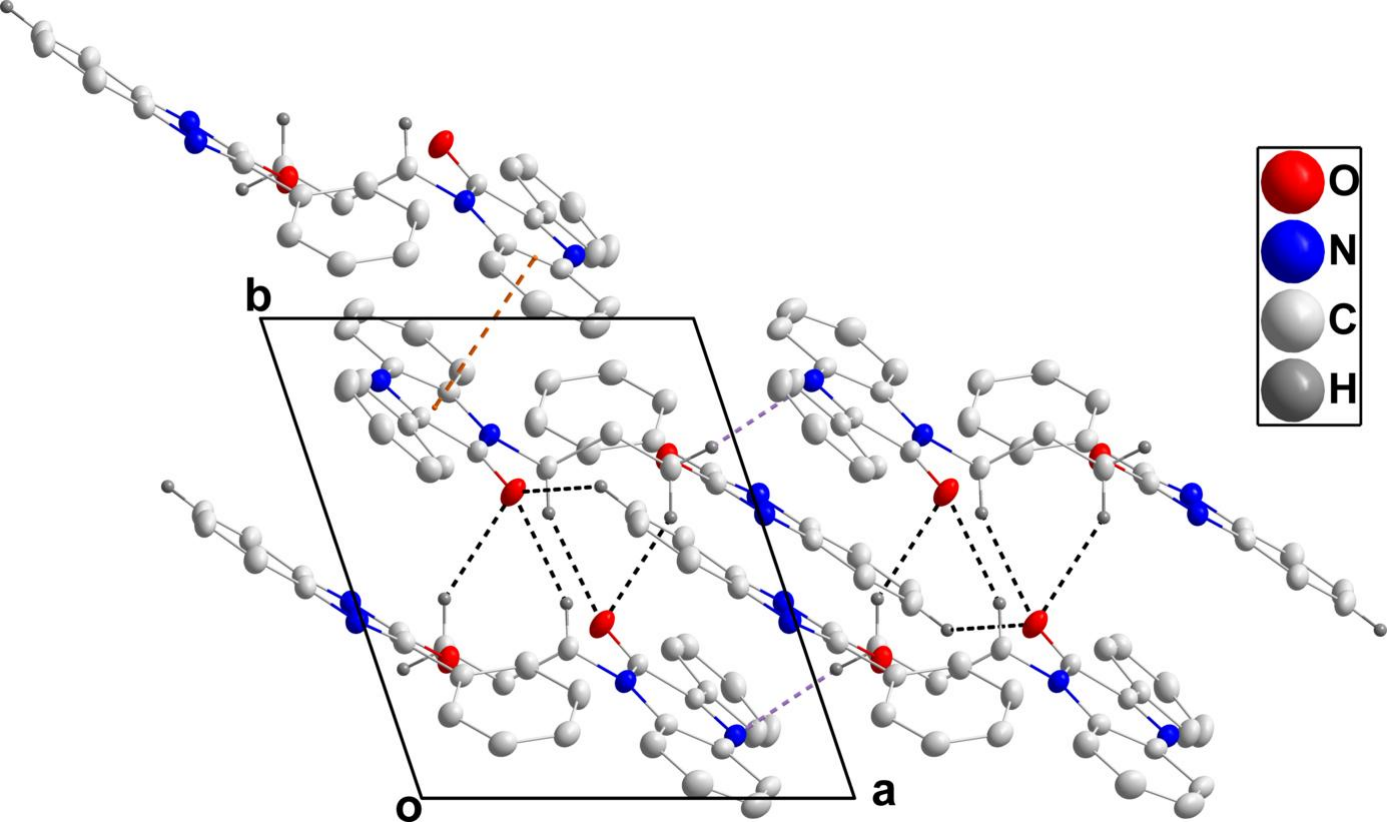
Packing viewed along the *c*-axis direction with inter­molecular inter­actions depicted as in Fig. 2 ▸.

**Table 1:** Hydrogen-bond geometry (Å, °)

*D*—H⋯*A*	*D*—H	H⋯*A*	*D*⋯*A*	*D*—H⋯*A*
C14—H14⋯O1	0.961 (14)	2.197 (13)	2.8225 (14)	121.7 (10)
C15—H15*B*⋯O1^i^	0.962 (12)	2.503 (12)	3.3484 (13)	146.6 (9)
C17—H17*A*⋯N1^ii^	0.976 (13)	2.581 (13)	3.5523 (14)	173.3 (10)
C17—H17*B*⋯O1^i^	1.021 (12)	2.568 (13)	3.4700 (15)	147.1 (9)
C21—H21⋯O1^iii^	0.971 (14)	2.404 (14)	3.2166 (15)	140.9 (11)

Symmetry codes: (i) 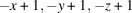 ; (ii) 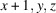 ; (iii) 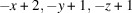 .

**Table 2:** Selected torsion angles (°)

C1—N2—C15—C16	101.04 (11)	C15—C16—C17—O2	68.47 (12)
N2—C15—C16—C17	178.73 (8)	C17—O2—C24—N3	−2.55 (14)
C24—O2—C17—C16	179.54 (8)		

**Table 3:** Selected geometrical parameters (Å, °) for related mol­ecules

Refcode	*d*_max_	r.m.s.d.	α*^*a*^*	N—C_α_—C_β_—*X^*b*^*
AZAZEC	0.031 (1)	0.001	13.25 (4)	−171.93 (8)
BUDMAP*^*c*^*	0.043 (1)	0.002	30.44 (7)	–
	0.023 (2)	0.002	19.31 (7)	–
ECUCOY	0.064 (1)	0.001	9.39 (6)	−76.77 (15)
IDOSUR	0.055 (2)	0.002	30.77 (8)	66.3 (2)
ESUKUB	0.030 (1)	0.001	12.04 (5)	−178.70 (9)
FOFCIQ	0.052 (1)	0.002	22.82 (10)	−115.4 (2)
ILIRED	0.030 (2)	0.002	18.75 (10)	179.86 (18)
NIBXEE*^*c*^*	0.038 (5)	0.002	28.4 (2)	156.3 (5)
	0.38 (5)	0.002	23.1 (2)	−154.2 (5)
PUGGII	0.035 (1)	0.002	28.39 (11)	178.12 (13)
RIRBOM	0.039 (1)	0.001	44.46 (4)	−168.64 (8)
UDAMIZ	0.060 (2)	0.002	20.39 (4)	171.2 (2)
UFITEM	0.063 (1)	0.001	34.67 (6)	176.19 (11)
XEXWIJ	0.022 (2)	0.002	19.63 (7)	−179.37 (14)
YAJGEX*^*c*^*	0.023 (1)	0.002	38.27 (10)	136.6 (2)
	0.037 (1)	0.002	37.14 (8)	−132.6 (2)

Notes: (*a*) Dihedral angle between mean planes of quinoxaline and attached phenyl rings; (*b*) torsion angle for first three atoms of chain attached to quinoxaline ring nitro­gen; (*c*) *Z* = 2.

**Table 4:** Experimental details

Crystal data	
Chemical formula	C_31_H_24_N_4_O_2_
*M*_r_	484.54
Crystal system, space group	Triclinic, *P* 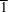
Temperature (K)	120
*a*, *b*, *c* (Å)	8.8038 (6), 10.2277 (7), 14.0937 (10)
α, β, γ (°)	90.262 (1), 96.630 (1), 108.395 (1)
*V* (Å^3^)	1195.00 (14)
*Z*	2
Radiation type	Mo *K*α
μ (mm^−1^)	0.09
Crystal size (mm)	0.41 × 0.34 × 0.10

Data collection	
Diffractometer	Bruker SMART APEX CCD
Absorption correction	Multi-scan (*SADABS*; Krause *et al.*, 2015 ▸)
*T*_min_, *T*_max_	0.84, 0.99
No. of measured, independent and observed [*I* > 2σ(*I*)] reflections	22697, 6292, 4672
*R*_int_	0.029
(sin θ/λ)_max_ (Å^−1^)	0.685

Refinement	
*R*[*F* ^2^ > 2σ(*F* ^2^)], *wR*(*F* ^2^), *S*	0.045, 0.129, 1.02
No. of reflections	6292
No. of parameters	430
H-atom treatment	All H-atom parameters refined
Δρ_max_, Δρ_min_ (e Å^−3^)	0.40, −0.22

Computer programs: *APEX3* and *SAINT* (Bruker, 2016 ▸), *SHELXT* (Sheldrick, 2015*a* ▸), *SHELXL2018/1* (Sheldrick, 2015*b* ▸), *DIAMOND* (Brandenburg & Putz, 2012 ▸) and *SHELXTL* (Sheldrick, 2008 ▸).

## supplementary crystallographic information

### Crystal data

**Table:**

C_31_H_24_N_4_O_2_	*Z* = 2
*M**_r_* = 484.54	*F*(000) = 508
Triclinic, *P*1	*D*_x_ = 1.347 Mg m^−^^3^
*a* = 8.8038 (6) Å	Mo *K*α radiation, λ = 0.71073 Å
*b* = 10.2277 (7) Å	Cell parameters from 8578 reflections
*c* = 14.0937 (10) Å	θ = 2.5–29.0°
α = 90.262 (1)°	µ = 0.09 mm^−^^1^
β = 96.630 (1)°	*T* = 120 K
γ = 108.395 (1)°	Plate, colourless
*V* = 1195.00 (14) Å^3^	0.41 × 0.34 × 0.10 mm

### Data collection

**Table:**

Bruker SMART APEX CCD diffractometer	6292 independent reflections
Radiation source: fine-focus sealed tube	4672 reflections with *I* > 2σ(*I*)
Graphite monochromator	*R*_int_ = 0.029
Detector resolution: 8.3333 pixels mm^-1^	θ_max_ = 29.1°, θ_min_ = 2.1°
φ and ω scans	*h* = −11→11
Absorption correction: multi-scan (*SADABS*; Krause *et al.*, 2015)	*k* = −13→13
*T*_min_ = 0.84, *T*_max_ = 0.99	*l* = −19→19
22697 measured reflections	

### Refinement

**Table:**

Refinement on *F*^2^	Primary atom site location: structure-invariant direct methods
Least-squares matrix: full	Secondary atom site location: difference Fourier map
*R*[*F*^2^ > 2σ(*F*^2^)] = 0.045	Hydrogen site location: difference Fourier map
*wR*(*F*^2^) = 0.129	All H-atom parameters refined
*S* = 1.01	*w* = 1/[σ^2^(*F*_o_^2^) + (0.0859*P*)^2^] where *P* = (*F*_o_^2^ + 2*F*_c_^2^)/3
6292 reflections	(Δ/σ)_max_ < 0.001
430 parameters	Δρ_max_ = 0.40 e Å^−^^3^
0 restraints	Δρ_min_ = −0.22 e Å^−^^3^

### Special details

**Table:**

Experimental. The diffraction data were obtained from 3 sets of 400 frames, each of width 0.5° in ω, colllected at φ = 0.00, 90.00 and 180.00° and 2 sets of 800 frames, each of width 0.45° in φ, collected at ω = –30.00 and 210.00°. The scan time was 15 sec/frame.
Geometry. All esds (except the esd in the dihedral angle between two l.s. planes) are estimated using the full covariance matrix. The cell esds are taken into account individually in the estimation of esds in distances, angles and torsion angles; correlations between esds in cell parameters are only used when they are defined by crystal symmetry. An approximate (isotropic) treatment of cell esds is used for estimating esds involving l.s. planes.
Refinement. Refinement of F^2^ against ALL reflections. The weighted R-factor wR and goodness of fit S are based on F^2^, conventional R-factors R are based on F, with F set to zero for negative F^2^. The threshold expression of F^2^ > 2sigma(F^2^) is used only for calculating R-factors(gt) etc. and is not relevant to the choice of reflections for refinement. R-factors based on F^2^ are statistically about twice as large as those based on F, and R- factors based on ALL data will be even larger.

### Fractional atomic coordinates and isotropic or equivalent isotropic displacement parameters (Å^2^)

**Table:**

	*x*	*y*	*z*	*U*_iso_*/*U*_eq_	
O1	0.44841 (10)	0.63740 (8)	0.58310 (5)	0.02992 (19)	
O2	0.83077 (9)	0.71124 (8)	0.31407 (5)	0.02503 (18)	
N1	0.22753 (10)	0.87022 (9)	0.52543 (6)	0.0235 (2)	
N2	0.43848 (10)	0.75671 (9)	0.44934 (6)	0.02157 (19)	
N3	1.01284 (10)	0.59246 (9)	0.31524 (6)	0.0239 (2)	
H3	0.3852 (17)	1.0016 (15)	0.1911 (11)	0.051 (4)*	
N4	1.01195 (11)	0.63096 (9)	0.11706 (6)	0.0258 (2)	
C1	0.37800 (12)	0.84656 (11)	0.39487 (7)	0.0229 (2)	
C2	0.42362 (15)	0.88805 (12)	0.30498 (8)	0.0301 (3)	
H2	0.5105 (16)	0.8643 (14)	0.2796 (9)	0.036 (3)*	
C3	0.35202 (16)	0.97177 (13)	0.25365 (9)	0.0362 (3)	
C4	0.23277 (16)	1.01518 (13)	0.28870 (9)	0.0349 (3)	
H4	0.1835 (16)	1.0778 (14)	0.2494 (10)	0.041 (4)*	
C5	0.19108 (14)	0.97842 (11)	0.37813 (8)	0.0287 (2)	
H5	0.1082 (17)	1.0078 (14)	0.4048 (9)	0.040 (4)*	
C6	0.26590 (12)	0.89688 (11)	0.43344 (7)	0.0233 (2)	
C7	0.29185 (12)	0.79293 (10)	0.57833 (7)	0.0211 (2)	
C8	0.39776 (12)	0.72245 (11)	0.53976 (7)	0.0217 (2)	
C9	0.25817 (12)	0.77893 (11)	0.67950 (7)	0.0229 (2)	
C10	0.17893 (14)	0.86482 (13)	0.71543 (8)	0.0313 (3)	
H10	0.1495 (15)	0.9312 (13)	0.6724 (9)	0.030 (3)*	
C11	0.14777 (16)	0.85979 (15)	0.80948 (9)	0.0374 (3)	
H11	0.0946 (18)	0.9227 (16)	0.8322 (11)	0.053 (4)*	
C12	0.19357 (14)	0.76913 (13)	0.87067 (8)	0.0329 (3)	
H12	0.1671 (15)	0.7634 (13)	0.9406 (9)	0.034 (3)*	
C13	0.27219 (13)	0.68479 (12)	0.83650 (8)	0.0290 (2)	
H13	0.3018 (16)	0.6197 (14)	0.8811 (10)	0.040 (4)*	
C14	0.30485 (13)	0.68925 (11)	0.74210 (8)	0.0253 (2)	
H14	0.3544 (16)	0.6243 (14)	0.7217 (9)	0.035 (3)*	
C15	0.54524 (12)	0.68814 (12)	0.41224 (8)	0.0232 (2)	
H15A	0.5299 (14)	0.6874 (12)	0.3405 (9)	0.025 (3)*	
H15B	0.5145 (14)	0.5943 (13)	0.4317 (8)	0.024 (3)*	
C16	0.72111 (12)	0.75993 (12)	0.45135 (8)	0.0239 (2)	
H16A	0.7551 (14)	0.8581 (14)	0.4330 (9)	0.032 (3)*	
H16B	0.7306 (14)	0.7557 (12)	0.5222 (9)	0.025 (3)*	
C17	0.83182 (13)	0.68895 (12)	0.41570 (7)	0.0247 (2)	
H17A	0.9421 (15)	0.7312 (12)	0.4463 (9)	0.028 (3)*	
H17B	0.7929 (14)	0.5849 (13)	0.4237 (8)	0.028 (3)*	
C18	1.10461 (13)	0.56007 (11)	0.16404 (8)	0.0253 (2)	
C19	1.19802 (14)	0.50343 (12)	0.11175 (9)	0.0306 (3)	
H19	1.1921 (16)	0.5151 (14)	0.0421 (10)	0.040 (4)*	
C20	1.28637 (14)	0.42787 (12)	0.15725 (9)	0.0334 (3)	
H20	1.3517 (17)	0.3897 (14)	0.1214 (10)	0.044 (4)*	
C21	1.28456 (13)	0.40650 (12)	0.25544 (9)	0.0330 (3)	
H21	1.3445 (16)	0.3504 (14)	0.2869 (9)	0.037 (3)*	
C22	1.19463 (13)	0.46076 (12)	0.30774 (9)	0.0294 (3)	
H22	1.1904 (15)	0.4469 (13)	0.3764 (9)	0.033 (3)*	
C23	1.10319 (12)	0.53882 (11)	0.26299 (8)	0.0243 (2)	
C24	0.92400 (12)	0.65636 (11)	0.26811 (7)	0.0221 (2)	
C25	0.91928 (12)	0.67574 (11)	0.16611 (7)	0.0228 (2)	
C26	0.81246 (13)	0.74470 (11)	0.11251 (7)	0.0234 (2)	
C27	0.65431 (13)	0.72358 (12)	0.13098 (8)	0.0288 (2)	
H27	0.6113 (15)	0.6636 (13)	0.1822 (9)	0.033 (3)*	
C28	0.55596 (14)	0.78379 (13)	0.07494 (8)	0.0315 (3)	
H28	0.4445 (16)	0.7639 (13)	0.0886 (9)	0.036 (3)*	
C29	0.61433 (14)	0.86632 (12)	0.00127 (8)	0.0300 (3)	
H29	0.5435 (15)	0.9110 (13)	−0.0411 (9)	0.034 (3)*	
C30	0.77094 (15)	0.88709 (12)	−0.01796 (8)	0.0300 (3)	
H30	0.8131 (15)	0.9449 (13)	−0.0704 (9)	0.035 (3)*	
C31	0.86847 (14)	0.82589 (12)	0.03672 (8)	0.0272 (2)	
H31	0.9791 (16)	0.8377 (13)	0.0246 (9)	0.032 (3)*	

### Atomic displacement parameters (Å^2^)

**Table:**

	*U*^11^	*U*^22^	*U*^33^	*U*^12^	*U*^13^	*U*^23^
O1	0.0366 (4)	0.0343 (4)	0.0290 (4)	0.0232 (4)	0.0106 (3)	0.0100 (3)
O2	0.0259 (4)	0.0336 (4)	0.0206 (4)	0.0158 (3)	0.0055 (3)	0.0026 (3)
N1	0.0238 (4)	0.0232 (5)	0.0249 (5)	0.0102 (4)	0.0006 (3)	−0.0003 (4)
N2	0.0226 (4)	0.0230 (4)	0.0222 (4)	0.0108 (3)	0.0052 (3)	0.0029 (3)
N3	0.0208 (4)	0.0257 (5)	0.0261 (5)	0.0087 (4)	0.0026 (3)	−0.0001 (4)
N4	0.0253 (5)	0.0282 (5)	0.0261 (5)	0.0112 (4)	0.0039 (4)	−0.0007 (4)
C1	0.0235 (5)	0.0211 (5)	0.0232 (5)	0.0066 (4)	0.0000 (4)	0.0020 (4)
C2	0.0317 (6)	0.0332 (6)	0.0269 (6)	0.0116 (5)	0.0053 (4)	0.0055 (5)
C3	0.0443 (7)	0.0375 (7)	0.0272 (6)	0.0140 (6)	0.0025 (5)	0.0085 (5)
C4	0.0446 (7)	0.0305 (6)	0.0305 (6)	0.0173 (5)	−0.0065 (5)	0.0041 (5)
C5	0.0323 (6)	0.0246 (6)	0.0305 (6)	0.0138 (5)	−0.0041 (5)	−0.0021 (4)
C6	0.0248 (5)	0.0200 (5)	0.0250 (5)	0.0084 (4)	−0.0011 (4)	−0.0005 (4)
C7	0.0189 (5)	0.0208 (5)	0.0246 (5)	0.0078 (4)	0.0026 (4)	−0.0004 (4)
C8	0.0209 (5)	0.0221 (5)	0.0240 (5)	0.0093 (4)	0.0045 (4)	0.0028 (4)
C9	0.0188 (5)	0.0246 (5)	0.0251 (5)	0.0062 (4)	0.0038 (4)	−0.0011 (4)
C10	0.0316 (6)	0.0372 (7)	0.0315 (6)	0.0191 (5)	0.0061 (5)	0.0015 (5)
C11	0.0356 (7)	0.0482 (8)	0.0361 (7)	0.0228 (6)	0.0086 (5)	−0.0046 (6)
C12	0.0295 (6)	0.0435 (7)	0.0264 (6)	0.0113 (5)	0.0072 (4)	−0.0031 (5)
C13	0.0293 (6)	0.0314 (6)	0.0263 (6)	0.0086 (5)	0.0059 (4)	0.0031 (5)
C14	0.0243 (5)	0.0260 (6)	0.0271 (5)	0.0091 (4)	0.0066 (4)	0.0015 (4)
C15	0.0226 (5)	0.0264 (6)	0.0241 (5)	0.0121 (4)	0.0050 (4)	0.0004 (4)
C16	0.0236 (5)	0.0272 (6)	0.0224 (5)	0.0097 (4)	0.0044 (4)	0.0005 (4)
C17	0.0222 (5)	0.0333 (6)	0.0206 (5)	0.0113 (4)	0.0040 (4)	0.0030 (4)
C18	0.0218 (5)	0.0258 (5)	0.0288 (6)	0.0085 (4)	0.0029 (4)	−0.0024 (4)
C19	0.0284 (6)	0.0344 (6)	0.0315 (6)	0.0131 (5)	0.0050 (5)	−0.0047 (5)
C20	0.0249 (5)	0.0343 (6)	0.0436 (7)	0.0140 (5)	0.0021 (5)	−0.0099 (5)
C21	0.0248 (6)	0.0311 (6)	0.0447 (7)	0.0143 (5)	−0.0043 (5)	−0.0045 (5)
C22	0.0247 (5)	0.0313 (6)	0.0329 (6)	0.0119 (5)	−0.0021 (4)	−0.0010 (5)
C23	0.0201 (5)	0.0238 (5)	0.0283 (5)	0.0070 (4)	0.0007 (4)	−0.0024 (4)
C24	0.0205 (5)	0.0236 (5)	0.0228 (5)	0.0071 (4)	0.0045 (4)	−0.0001 (4)
C25	0.0223 (5)	0.0234 (5)	0.0229 (5)	0.0074 (4)	0.0034 (4)	0.0006 (4)
C26	0.0267 (5)	0.0247 (5)	0.0206 (5)	0.0109 (4)	0.0023 (4)	−0.0012 (4)
C27	0.0269 (5)	0.0351 (6)	0.0255 (6)	0.0111 (5)	0.0037 (4)	0.0047 (5)
C28	0.0259 (6)	0.0412 (7)	0.0299 (6)	0.0146 (5)	0.0021 (4)	0.0025 (5)
C29	0.0350 (6)	0.0307 (6)	0.0269 (6)	0.0158 (5)	−0.0012 (4)	−0.0004 (5)
C30	0.0384 (6)	0.0290 (6)	0.0245 (5)	0.0128 (5)	0.0052 (5)	0.0030 (5)
C31	0.0298 (6)	0.0293 (6)	0.0254 (5)	0.0123 (5)	0.0065 (4)	0.0017 (4)

### Geometric parameters (Å, º)

**Table:**

O1—C8	1.2309 (12)	C13—H13	0.991 (13)
O2—C24	1.3484 (12)	C14—H14	0.961 (13)
O2—C17	1.4509 (12)	C15—C16	1.5238 (15)
N1—C7	1.3007 (12)	C15—H15A	1.005 (12)
N1—C6	1.3841 (13)	C15—H15B	0.962 (12)
N2—C8	1.3804 (13)	C16—C17	1.5119 (15)
N2—C1	1.3922 (12)	C16—H16A	0.997 (13)
N2—C15	1.4748 (13)	C16—H16B	0.995 (12)
N3—C24	1.2987 (12)	C17—H17A	0.976 (13)
N3—C23	1.3722 (13)	C17—H17B	1.021 (12)
N4—C25	1.3102 (13)	C18—C19	1.4113 (15)
N4—C18	1.3721 (13)	C18—C23	1.4132 (15)
C1—C2	1.4005 (15)	C19—C20	1.3720 (16)
C1—C6	1.4057 (15)	C19—H19	0.987 (14)
C2—C3	1.3766 (16)	C20—C21	1.4031 (18)
C2—H2	0.976 (14)	C20—H20	0.972 (15)
C3—C4	1.3983 (18)	C21—C22	1.3735 (16)
C3—H3	0.980 (15)	C21—H21	0.972 (13)
C4—C5	1.3743 (17)	C22—C23	1.4057 (14)
C4—H4	1.014 (13)	C22—H22	0.982 (13)
C5—C6	1.4040 (14)	C24—C25	1.4497 (14)
C5—H5	0.982 (14)	C25—C26	1.4873 (14)
C7—C9	1.4881 (15)	C26—C31	1.3946 (16)
C7—C8	1.4907 (14)	C26—C27	1.3955 (15)
C9—C14	1.3964 (14)	C27—C28	1.3913 (15)
C9—C10	1.4068 (15)	C27—H27	0.980 (13)
C10—C11	1.3822 (17)	C28—C29	1.3828 (17)
C10—H10	0.987 (12)	C28—H28	0.979 (13)
C11—C12	1.3889 (18)	C29—C30	1.3862 (17)
C11—H11	0.979 (16)	C29—H29	1.025 (13)
C12—C13	1.3803 (16)	C30—C31	1.3834 (15)
C12—H12	1.036 (13)	C30—H30	0.981 (13)
C13—C14	1.3912 (15)	C31—H31	0.978 (13)
			
C24—O2—C17	116.19 (8)	C17—C16—C15	111.90 (9)
C7—N1—C6	120.18 (9)	C17—C16—H16A	109.3 (7)
C8—N2—C1	122.21 (8)	C15—C16—H16A	110.2 (7)
C8—N2—C15	115.91 (8)	C17—C16—H16B	107.8 (7)
C1—N2—C15	121.84 (8)	C15—C16—H16B	107.8 (7)
C24—N3—C23	116.53 (9)	H16A—C16—H16B	109.8 (10)
C25—N4—C18	118.08 (9)	O2—C17—C16	106.67 (8)
N2—C1—C2	123.22 (10)	O2—C17—H17A	107.9 (7)
N2—C1—C6	117.35 (9)	C16—C17—H17A	110.4 (7)
C2—C1—C6	119.43 (10)	O2—C17—H17B	107.4 (7)
C3—C2—C1	119.53 (11)	C16—C17—H17B	112.7 (7)
C3—C2—H2	118.9 (7)	H17A—C17—H17B	111.5 (10)
C1—C2—H2	121.5 (7)	N4—C18—C19	119.25 (10)
C2—C3—C4	121.45 (11)	N4—C18—C23	121.00 (9)
C2—C3—H3	119.3 (8)	C19—C18—C23	119.71 (10)
C4—C3—H3	119.2 (8)	C20—C19—C18	119.79 (11)
C5—C4—C3	119.29 (11)	C20—C19—H19	121.9 (8)
C5—C4—H4	121.2 (8)	C18—C19—H19	118.2 (8)
C3—C4—H4	119.4 (8)	C19—C20—C21	120.47 (11)
C4—C5—C6	120.49 (11)	C19—C20—H20	119.8 (8)
C4—C5—H5	121.1 (8)	C21—C20—H20	119.7 (8)
C6—C5—H5	118.4 (8)	C22—C21—C20	120.72 (11)
N1—C6—C5	118.29 (9)	C22—C21—H21	119.1 (8)
N1—C6—C1	122.12 (9)	C20—C21—H21	120.1 (8)
C5—C6—C1	119.59 (10)	C21—C22—C23	120.01 (11)
N1—C7—C9	117.64 (9)	C21—C22—H22	122.5 (7)
N1—C7—C8	121.39 (9)	C23—C22—H22	117.4 (7)
C9—C7—C8	120.97 (9)	N3—C23—C22	119.95 (10)
O1—C8—N2	119.63 (9)	N3—C23—C18	120.75 (9)
O1—C8—C7	124.16 (9)	C22—C23—C18	119.30 (10)
N2—C8—C7	116.22 (8)	N3—C24—O2	120.23 (9)
C14—C9—C10	117.79 (10)	N3—C24—C25	123.76 (9)
C14—C9—C7	124.24 (9)	O2—C24—C25	116.00 (9)
C10—C9—C7	117.93 (9)	N4—C25—C24	119.73 (9)
C11—C10—C9	120.94 (11)	N4—C25—C26	117.14 (9)
C11—C10—H10	120.7 (7)	C24—C25—C26	123.13 (9)
C9—C10—H10	118.3 (7)	C31—C26—C27	118.68 (10)
C10—C11—C12	120.62 (11)	C31—C26—C25	118.79 (10)
C10—C11—H11	118.5 (9)	C27—C26—C25	122.38 (10)
C12—C11—H11	120.9 (9)	C28—C27—C26	120.18 (11)
C13—C12—C11	119.04 (11)	C28—C27—H27	119.9 (7)
C13—C12—H12	120.4 (7)	C26—C27—H27	119.9 (7)
C11—C12—H12	120.6 (7)	C29—C28—C27	120.44 (11)
C12—C13—C14	120.89 (11)	C29—C28—H28	121.7 (7)
C12—C13—H13	117.6 (8)	C27—C28—H28	117.8 (7)
C14—C13—H13	121.5 (8)	C28—C29—C30	119.74 (10)
C13—C14—C9	120.71 (10)	C28—C29—H29	121.6 (7)
C13—C14—H14	116.8 (8)	C30—C29—H29	118.6 (7)
C9—C14—H14	122.4 (8)	C31—C30—C29	120.03 (11)
N2—C15—C16	111.24 (9)	C31—C30—H30	119.7 (7)
N2—C15—H15A	108.8 (7)	C29—C30—H30	120.3 (7)
C16—C15—H15A	110.8 (7)	C30—C31—C26	120.91 (11)
N2—C15—H15B	108.6 (7)	C30—C31—H31	122.1 (7)
C16—C15—H15B	108.8 (7)	C26—C31—H31	117.0 (7)
H15A—C15—H15B	108.5 (10)		
			
C8—N2—C1—C2	176.79 (10)	N2—C15—C16—C17	178.73 (8)
C15—N2—C1—C2	−5.61 (16)	C24—O2—C17—C16	179.54 (8)
C8—N2—C1—C6	−3.17 (15)	C15—C16—C17—O2	68.47 (12)
C15—N2—C1—C6	174.43 (9)	C25—N4—C18—C19	176.17 (10)
N2—C1—C2—C3	176.76 (10)	C25—N4—C18—C23	−1.52 (15)
C6—C1—C2—C3	−3.29 (17)	N4—C18—C19—C20	−177.53 (10)
C1—C2—C3—C4	−0.84 (19)	C23—C18—C19—C20	0.19 (17)
C2—C3—C4—C5	2.87 (19)	C18—C19—C20—C21	−0.02 (18)
C3—C4—C5—C6	−0.71 (18)	C19—C20—C21—C22	−0.04 (18)
C7—N1—C6—C5	178.67 (9)	C20—C21—C22—C23	−0.08 (18)
C7—N1—C6—C1	−2.17 (15)	C24—N3—C23—C22	−176.01 (9)
C4—C5—C6—N1	175.80 (10)	C24—N3—C23—C18	3.45 (15)
C4—C5—C6—C1	−3.38 (16)	C21—C22—C23—N3	179.71 (10)
N2—C1—C6—N1	6.18 (15)	C21—C22—C23—C18	0.24 (16)
C2—C1—C6—N1	−173.78 (10)	N4—C18—C23—N3	−2.09 (16)
N2—C1—C6—C5	−174.66 (9)	C19—C18—C23—N3	−179.76 (9)
C2—C1—C6—C5	5.38 (15)	N4—C18—C23—C22	177.37 (9)
C6—N1—C7—C9	174.31 (9)	C19—C18—C23—C22	−0.30 (16)
C6—N1—C7—C8	−4.74 (15)	C23—N3—C24—O2	179.55 (9)
C1—N2—C8—O1	176.52 (9)	C23—N3—C24—C25	−1.49 (15)
C15—N2—C8—O1	−1.21 (14)	C17—O2—C24—N3	−2.55 (14)
C1—N2—C8—C7	−3.15 (14)	C17—O2—C24—C25	178.42 (9)
C15—N2—C8—C7	179.12 (9)	C18—N4—C25—C24	3.46 (15)
N1—C7—C8—O1	−172.29 (10)	C18—N4—C25—C26	−176.34 (9)
C9—C7—C8—O1	8.70 (16)	N3—C24—C25—N4	−2.08 (16)
N1—C7—C8—N2	7.37 (15)	O2—C24—C25—N4	176.92 (9)
C9—C7—C8—N2	−171.65 (9)	N3—C24—C25—C26	177.71 (10)
N1—C7—C9—C14	173.29 (10)	O2—C24—C25—C26	−3.30 (15)
C8—C7—C9—C14	−7.66 (16)	N4—C25—C26—C31	−35.53 (14)
N1—C7—C9—C10	−8.94 (15)	C24—C25—C26—C31	144.68 (10)
C8—C7—C9—C10	170.12 (10)	N4—C25—C26—C27	139.95 (11)
C14—C9—C10—C11	−0.34 (18)	C24—C25—C26—C27	−39.84 (15)
C7—C9—C10—C11	−178.26 (11)	C31—C26—C27—C28	−0.54 (16)
C9—C10—C11—C12	−0.3 (2)	C25—C26—C27—C28	−176.02 (10)
C10—C11—C12—C13	0.6 (2)	C26—C27—C28—C29	−0.78 (17)
C11—C12—C13—C14	−0.34 (18)	C27—C28—C29—C30	1.20 (18)
C12—C13—C14—C9	−0.27 (18)	C28—C29—C30—C31	−0.29 (17)
C10—C9—C14—C13	0.60 (16)	C29—C30—C31—C26	−1.05 (17)
C7—C9—C14—C13	178.38 (10)	C27—C26—C31—C30	1.45 (17)
C8—N2—C15—C16	−81.23 (11)	C25—C26—C31—C30	177.10 (10)
C1—N2—C15—C16	101.04 (11)		

### Hydrogen-bond geometry (Å, º)

**Table:**

*D*—H···*A*	*D*—H	H···*A*	*D*···*A*	*D*—H···*A*
C14—H14···O1	0.961 (14)	2.197 (13)	2.8225 (14)	121.7 (10)
C15—H15*B*···O1^i^	0.962 (12)	2.503 (12)	3.3484 (13)	146.6 (9)
C17—H17*A*···N1^ii^	0.976 (13)	2.581 (13)	3.5523 (14)	173.3 (10)
C17—H17*B*···O1^i^	1.021 (12)	2.568 (13)	3.4700 (15)	147.1 (9)
C21—H21···O1^iii^	0.971 (14)	2.404 (14)	3.2166 (15)	140.9 (11)

Symmetry codes: (i) −*x*+1, −*y*+1, −*z*+1; (ii) *x*+1, *y*, *z*; (iii) −*x*+2, −*y*+1, −*z*+1.
